# IL-17A Damages the Blood–Retinal Barrier through Activating the Janus Kinase 1 Pathway

**DOI:** 10.3390/biomedicines9070831

**Published:** 2021-07-16

**Authors:** Eimear M. Byrne, María Llorián-Salvador, Miao Tang, Andriana Margariti, Mei Chen, Heping Xu

**Affiliations:** The Wellcome-Wolfson Institute for Experimental Medicine, School of Medicine, Dentistry and Biomedical Sciences, Queen’s University Belfast, Belfast BT9 7BL, UK; ebyrne19@qub.ac.uk (E.M.B.); m.lloriansalvador@qub.ac.uk (M.L.-S.); mtang01@qub.ac.uk (M.T.); a.margariti@qub.ac.uk (A.M.); m.chen@qub.ac.uk (M.C.)

**Keywords:** interleukin-17, blood–retinal barrier, retinopathy, macular oedema, inflammation, retina, JAK/STAT signaling, Tofacitinib Citrate

## Abstract

Blood–retinal barrier (BRB) dysfunction underlies macular oedema in many sight-threatening conditions, including diabetic macular oedema, neovascular age-related macular degeneration and uveoretinitis. Inflammation plays an important role in BRB dysfunction. This study aimed to understand the role of the inflammatory cytokine IL-17A in BRB dysfunction and the mechanism involved. Human retinal pigment epithelial (RPE) cell line ARPE19 and murine brain endothelial line bEnd.3 were cultured on transwell membranes to model the outer BRB and inner BRB, respectively. IL-17A treatment (3 days in bEnd.3 cells and 6 days in ARPE19 cells) disrupted the distribution of claudin-5 in bEnd.3 cells and ZO-1 in ARPE19 cells, reduced the transepithelial/transendothelial electrical resistance (TEER) and increased permeability to FITC-tracers in vitro. Intravitreal (20 ng/1 μL/eye) or intravenous (20 ng/g) injection of recombinant IL-17A induced retinal albumin leakage within 48 h in C57BL/6J mice. Mechanistically, IL-17A induced Janus kinase 1 (JAK1) phosphorylation in bEnd.3 but not ARPE19 cells. Blocking JAK1 with Tofacitinib prevented IL-17A-mediated claudin-5 dysmorphia in bEnd.3 cells and reduced albumin leakage in IL-17A-treated mice. Our results suggest that IL-17A can damage the BRB through the activating the JAK1 signaling pathway, and targeting this pathway may be a novel approach to treat inflammation-induced macular oedema.

## 1. Introduction

The blood–retinal barrier (BRB) segregates the neuroretina from the periphery, thereby protecting the retina from exogenous pathogen invasion and systemic inflammatory disturbances. The BRB consists of the inner BRB (iBRB), i.e., tight junctions between retinal endothelial cells and outer BRB (oBRB), i.e., tight junctions between retinal pigment epithelial (RPE) cells. BRB dysfunction may result in the infiltration of circulating immune cells and the leakage of fluid into the neuroretina, causing retinal oedema. When oedema occurs in the macula (known as macular oedema), it can severely damage visual function. BRB dysfunction underpins many sight-threatening retinal diseases, such as diabetic macular oedema (DMO) [1], age-related macular degeneration (AMD) [2,3] and uveitis [4,5].

The mechanisms underpinning BRB dysfunction are complicated and incompletely understood. Inflammation is known to play an important role, and intravitreal injection of steroids, such as triamcinolone [6,7,8,9] and intravitreal steroid implants (e.g., Ozurdex [10] and Retisert [11,12,13]), has proven to be effective in controlling macular oedema caused by diabetic retinopathy (DR) or uveitis. However, these therapies often cause severe adverse effects, such as steroid-induced glaucoma and cataract [14,15,16]. Abnormal levels of vascular endothelial growth factor (VEGF) critically contribute to BRB leakage [17], and intravitreal injection of VEGF inhibitors (e.g., anti-VEGF neutralizing antibodies) is the standard of care for macular oedema in various conditions (e.g., DR, neovascular AMD or uveitis) [18,19,20]. A significant proportion of patients, however, are refractory to VEGF-targeted therapies [20,21,22]. Furthermore, since VEGF is an essential growth factor for neurons, sustained depletion of intraocular VEGF [23] or VEGF family members [24] can accelerate retinal neurodegeneration in diseased eyes [25]. These factors highlight the urgent need for more effective and safe therapies for patients with macular oedema caused by various pathogenic factors.

Inflammation damages the BRB by releasing various inflammatory mediators, such as IL-1β, IL-8, IL-17A, IL-6, MCP-1 and VEGF. Higher levels of inflammatory cytokines and chemokines have been reported in the plasma and intraocular fluids in DR [26,27,28] and neovascular AMD patients [29,30,31]. Among various cytokines, we are particularly interested in the role IL-17A in BRB dysfunction for two reasons. Firstly, IL-17A is critically involved in barrier dysfunction in the skin [32], the gastrointestinal barrier [33] and blood–brain barrier [34]. Secondly, IL-17A critically contributes to the pathogenesis of DR [35,36,37,38], AMD [39,40] and uveitis [41,42,43], all of which can lead to macular oedema.

The levels of IL-17A in the vitreous fluid and serum of proliferative DR patients were increased compared to the controls [44]. Additionally, an animal study has shown that treatment with IL-17A antibody ameliorates DR pathology [37]. IL-17A knockout mice have been found to have less severe disease phenotype than wild-type mice in experimental autoimmune uveoretinitis (EAU) [45], and IL-17A is elevated in the serum of uveitic patients [42]. Higher levels of IL-17A were also observed in the serum of AMD patients [46], and IL-17A was detected in the macula of both neovascular and geographic atrophy AMD patients [47].

In this study, we found that IL-17A could induce alterations in tight junction structure and impair both iBRB and oBRB function. Furthermore, we found that IL-17A-induced iBRB dysfunction is mediated by the Janus kinase 1 (JAK1) signaling pathway and that JAK1 inhibitor Tofacitinib Citrate can effectively suppress IL-17A-induced iBRB leakage. Our results suggest that the JAK/STAT pathway is an attractive therapeutic target for IL-17A-induced macular oedema.

## 2. Materials and Methods

### 2.1. Cell Culture

The human RPE cell line ARPE-19 (ATCC CRL-2302™, Manassas, VA, USA) cells were cultured in Dulbecco’s Modified Eagle Medium: Nutrient Mixture F-12 (DMEM/F12) (Gibco, Catalog No. 11320033) supplemented with 10% FCS (Gibco™ Catalog No. 10270106) and 1% penicillin–streptomycin (Gibco, Catalog No. 15140122) (all from. Gibco, Waltham, MA, USA). For experiments, media were changed to lower serum (1% FCS), to facilitate RPE cell quiescence. The mouse-brain endothelial cell line bEnd.3 cells (ATCC^®^ CRL-2299™) were cultured and maintained for experiments in Dulbecco’s Modified Eagle Medium with GlutaMAX™ Supplement (Gibco, Catalogue No. 10566016) medium supplemented with 10% FCS and 1% penicillin–streptomycin of same origins as above.

### 2.2. Transwell Cell Culture

Corning PET transwell inserts (6.5 mm diameter, 0.33 cm^2^ area, 0.4 µm pore size, Corning, Catalog no.3413 Corning, New York, NY, USA) were used for all experiments. For ARPE-19 cells, inserts were coated with 2% Geltrex^®^ LDEV-Free hESC-qualified Reduced Growth Factor Basement Membrane Matrix (Gibco) prior to cell seeding. ARPE-19 cells were seeded at a density of 30,000 cells/insert in quiescence media and cultured for 3 weeks, with media changes twice weekly before experiments. The bEnd.3 cells were seeded onto transwells directly, at a density of 50,000 cells/insert, in normal culture media, and grown for 3 days before experiments.

### 2.3. Cell Treatments

ARPE-19 cells were treated with human recombinant IL-17A (Catalog No. 7955-IL/CF, R&D Systems, Abingdon, UK) for 6 days of treatment. Overall, 50 ng/mL IL-17A was sufficient for tight junction dysmorphia and barrier dysfunction [48]. Then 30 min treatment with the same dose was used to examine JAK1 phosphorylation in response to IL-17A.

The bEnd.3 cells were treated with murine recombinant IL-17A (R&D Systems, Catalog No. 7956-ML-025/CF), at a concentration of 100 ng/mL. Three days of treatment with 100 ng/mL IL-17A was sufficient for tight junction dysmorphia and barrier dysfunction [49]. Thirty minutes of treatment with the same dose was used to examine JAK1 phosphorylation in response to IL-17A. Tofacitinib Citrate (Catalogue No. PZ0017, Sigma-Aldrich, St. Louis, MO, USA) (25 mg) was dissolved in 100 µL DMSO and further diluted in PBS, immediately before use, to 2.5 ug/mL (4.955 µM). Vehicle control for Tofacitinib was DMSO diluted 1:100,000 in PBS, i.e., 0.00001% DMSO.

Cell viability was assessed by using AlamarBlue™ Cell Viability Reagent (Thermo Fisher, Catalog No. DAL1100, Thermo Fisher, Waltham, MA, USA). Cells were regularly screened for mycoplasma, using LookOut^®^ Mycoplasma PCR Detection Kit (Catalog No. MP0035, Sigma-Aldrich).

### 2.4. Transepithelial Electrical Resistance (TEER)

The effects of IL-17A on TEER of both bEnd.3 and ARPE-19 cells were measured by using an EVOM2 Volt/Ohm meter and STX electrodes (World Precision Instruments, Sarasota, FL, USA). TEER values were measured from each of the three holes in the inserts, which were averaged for each well. Background TEER values were calculated by measuring values from wells with no cells. Final TEER values were calculated according to the following formula:

TEER (ohm/cm^2^) = [(Average value of 3 measures per well) − (Empty insert TEER)] × (Area of Insert)

### 2.5. FITC-Permeability Assay

FITC-Dextran 4kDa (Catalog No. 46944, Sigma-Aldrich) and FITC-Na (Catalog No. F6377, Sigma-Aldrich) were used for permeability studies in ARPE-19 [48] and bEnd.3 [50] as described previously, following IL-17A treatment. Then 200 µL FITC-Dextran or FITC-Na (1 mg/mL) diluted in media was added to the apical chamber of transwell inserts. Fluorescence intensity of apical and basal chambers were measured at 495–570 nm by using a POLARstar Omega plate reader (BMG Labtech, Baden-Wurttemgerg, Germany). FITC concentrations were interpolated from the standard curve. Diffusion rate calculation was adapted from a previously published formula [48].

Diffusion rate (%) = (FITC concentration in basal chamber × 100)/(FITC concentration in apical chamber)

### 2.6. Immunocytochemistry

Following IL-17A treatment, with or without Tofacitinib Citrate, ARPE-19 and bEnd.3 cells were stained for rabbit anti-ZO-1 and rabbit anti-claudin-5, respectively, to examine tight junction alterations. Both cell types were stained with Rabbit Phospho-JAK1 (Tyr1034, Tyr1035) antibody to examine JAK1 phosphorylation, following IL-17A treatment. All the above antibodies were from Thermo Fisher. Antibody details are shown in the Table 1.

### 2.7. Animal Care and Housing

*C57BL6/J* mice aged 2 months old, of both sexes, were used for these studies; *n* ≥ 5 animals were assigned per experimental group. Mice were maintained in the Biological Services Unit at Queen’s University Belfast, with free access to food and water on a 12 h light/dark cycle, in accordance with the ARVO Statement for the Use of Animals in Ophthalmic and Vision Research. All procedures were approved by the UK Home Office Animals (Scientific Procedures) Act 1986 and the local animal welfare ethical review board of Queen’s University Belfast (DOH PPL2876).

### 2.8. IL-17A Intravitreal and Intravenous Injection

**Figure A1:**
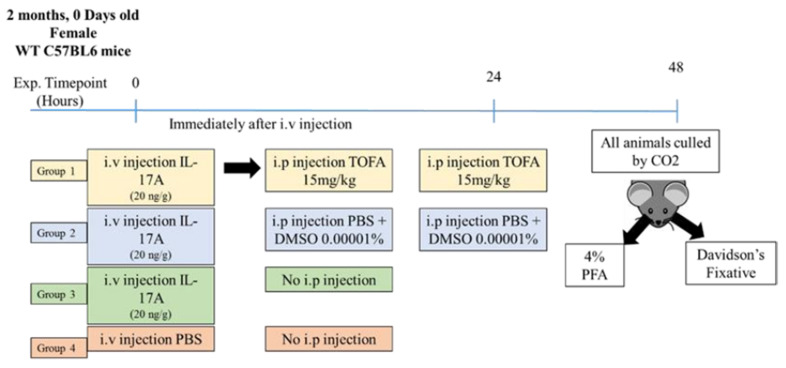
Study design: effect of Tofacitinib Citrate on IL-17A-mediated BRB leakage in WT mice.

Two-month-old C57BL/6J mice were injected intravitreally (ivt, 20 ng/μL/eye, *n* = 20 mice animals per group) or intravenously (i.v. 20 ng/g, *n* = 6 animals per group) with murine recombinant IL-17A (R&D Systems). Forty-eight hours after, animals were subjected to Micron IV examination, and eyes were collected for immunostaining or retinal protein was extracted for Western blot (see details below). For Tofacitinib Citrate treatment, 2-month-old C57BL6/J mice were treated with Tofacitinib Citrate (15 mg/kg, i.p.) immediately after IL-17A i.v. injection. Tofacitinib Citrate was administered again 24 h later, and mice were culled at 48 h (Appendix A Figure A1). Eyes were processed for Western blot for albumin (marker of BRB leakage) and immunostaining for pJAK1 and albumin.

### 2.9. In Vivo Fundus Imaging Using Micron IV

Mouse pupils were dilated by using atropine (1% *w*/*v*) and phenylephrine hydrochloride (2.5% *w*/*v*) (Bausch + Lomb, address United Kingdom), and the ocular surface was moistened with Viscotears (Novartis Pharmaceuticals Ltd., Surrey, UK). Mice were anaesthetized with ketamine hydrochloride (60 mg/kg, Fort George Animal Centre, Southampton, UK) and xylazine (5 mg/kg, Pharmacia & Veterinary Products, Kiel, Germany) via intraperitoneal injection. Fundus fluorescence angiography images FFA images were taken 2 min after intravenous injection of 100 µL of 1 mg/mL 4kDa FITC-Dextran, using Micron IV (Phoenix Technology Group, Pleasanton, CA, USA).

### 2.10. Albumin Leakage Quantification

Paraffin-embedded eyes were sectioned at 5 µm thickness. De-waxing was carried out by immersing slides in 3 changes of clearene for 5 min each, followed by 3 changes of 100% ethanol for 3 min each, and followed by 5 min in running water. Citraconic anhydride (Sigma, Catalog No. 125318), pH 7.4, at 95 °C, for 30 min, was used for antigen retrieval. Slides were incubated with goat anti-albumin and Biotinylated Griffonia Simplicifolia Lectin I Isolectin B4 (Table 1), overnight, at 4 °C. The next day, slides were washed in PBS prior to incubation with appropriate secondary antibodies. Slides were mounted with DAPI-Vectashield and imaged by using a Leica DMi8 epifluorescence microscope. Images were analyzed by using FIJI (NIH); Isolectin B4-positive ROIs were restored on the albumin channel and measured prior to whole neuroretina measurements. Leakage ratio was calculated as follows:

Leakage Ratio = (Extravascular albumin)/(Total albumin in neuroretina)

### 2.11. pJAK1 Quantification Neuroretina

Paraffin-embedded eyes were treated as above. After blocking, pJAK1 antibody was applied at a concentration of 1:100, at 4 °C, overnight. After several washes, secondary antibody donkey anti-rabbit 594 (Stratech Scientific Ltd, Ely, UK) was applied at 1:300 for 2 h. Slides were washed, mounted with DAPI-vectashield and imaged as above. Then pJAK1 in the neuroretina was quantified by using FIJI.

### 2.12. Western Blot

Samples were homogenized in RIPA buffer containing protease inhibitor cocktail (Sigma) and PhosSTOP™ (Roche). Protein concentrations were normalized by using Pierce BCA protein assay kit (Thermo Fisher, Catalog# 23225). Protein samples of equal concentrations were denatured by using NuPAGE™ 10X Reducer and LDS Sample Buffer (4X) (Thermo Fisher, Catalog No. NP0004, NP0007) as per the manufacturer’s instructions. Then 10 µg (retinal protein for albumin detection) or 35 µg (cell protein for phospho-JAK1 detection) was loaded on 10% acrylamide gels. Proteins were transferred to PVDF membranes (Immun-Blot, Bio-Rad Laboratory, Hercules, CA, USA), using the wet transfer method. Membranes were blocked with 5% Bovine Serum Albumin (Sigma-Aldrich, Catalog No. A3803) for 1 h, before primary antibody incubation overnight. The next day, membranes were washed, incubated in appropriate secondary antibody washed and developed by using Clarity Western ECL Blotting Substrate (Bio-Rad, Catalog No. 1705061). Membranes were imaged by using G:BOX Chemi XRQ chemiluminescence imager (Syngene, Cambridge, UK). Secondary antibody probing was used to confirm successful stripping. Densitometry was analyzed by using FIJI software.

### 2.13. Statistical Analysis

Graph generation and statistical analyses were performed by using GraphPad Prism 9 (GraphPad Software Inc. San Diego, CA, USA). The differences between two groups were compared by using unpaired *t*-test. For the difference between three or more groups, we used One-Way ANOVA, followed by Tukey’s (to compare the means of each groups with the means of every other group) or Dunnett’s (to compare the means of each treatment group with the mean on of the control group) multiple comparison post hoc tests.

## 3. Results

### 3.1. The Effect of IL-17A on bEnd3 Cell and ARPE19 Cell Tight Junctions and Barrier Function

**Figure 1:**
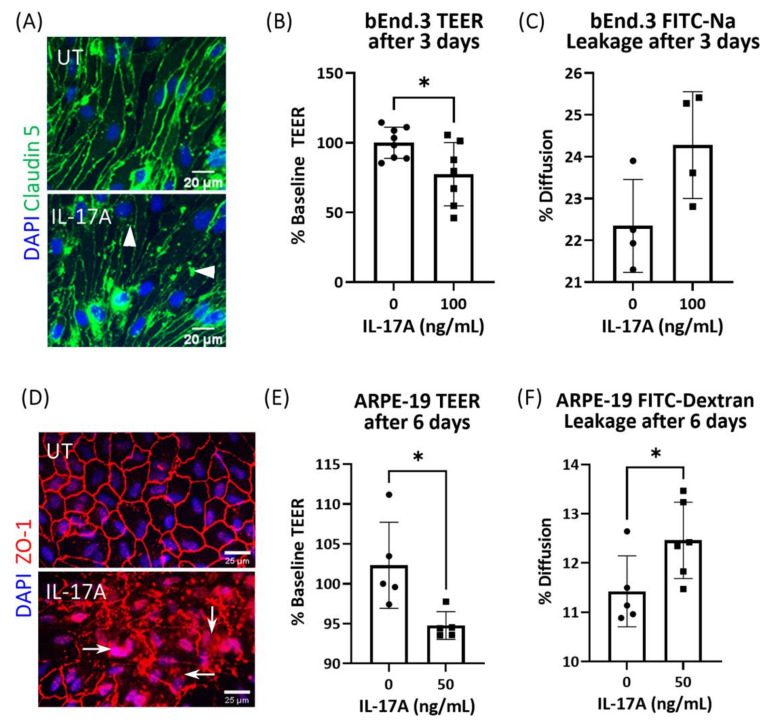
The effect of IL-17A on bEnd.3 cell and ARPE19 cell tight junctions and barrier function. Tight junction morphology and barrier function in bEnd.3 and ARPE19 cells were assessed after IL-17A treatment. (**A**) Immunostaining for claudin-5 (green) and DAPI (blue) in bEnd.3 cells after treatment with IL-17A (100 ng/mL) for 72 h. Scale bar = 20 µm. (**B**) TEER and (**C**) FITC-Na leakage of bEnd.3 cells cultured in transwell membrane after 3 days of treatment with IL-17A (100 ng/mL). (**D**) Immunostaining for ZO-1 (red) and DAPI (blue) in ARPE-19 cells after 6 days of treatment with IL-17A (50 ng/mL). Scale bar = 25 µm. (**E**) TEER and (**F**) FITC-Dextran permeability of ARPE-19 cells cultured in transwell membrane after 6 days of treatment with IL-17A (50 ng/mL). Mean ± SD; * *p* < 0.05 by unpaired *t*-test.

Claudin-5 junctions are integral to endothelial cell tight junctions. Under normal culture conditions, claudin-5 was evenly distributed around the cell–cell junction of bEnd3 cells (Figure 1A). IL-17A treatment (72 h) induced fragmented and clustered claudin-5 expression in bEnd.3 cells (arrowheads, Figure 1A). Measurement of trans-epithelial electrical resistance (TEER) in bEnd3 cells cultured in the transwell membrane showed that 72 h of treatment with IL-17A significantly reduced TEER (Figure 1B). FITC-Na permeability showed a pattern indicative of leakage, but was not statistically significant in IL-17A-treated bEnd3 cells (Figure 1C).

A previous study reported ZO-1 junction dysmorphia in ARPE-19 cells following 6 days of IL-17A treatment [48]. This was confirmed in our study (Figure 1D). Six days of IL-17A treatment induced fragmented ZO-1 junctions and positive staining in the cytosol and nuclei in ARPE-19 cells (arrows, Figure 1D). The treatment also significantly reduced TEER (Figure 1E) and increased FITC-Dextran (4 kDa) permeability (Figure 1F) in ARPE19 cells cultured in transwell membranes. Together, these data suggest that IL-17A alone can induce both vascular endothelial cell (iBRB) and retinal pigment epithelial cell (oBRB) dysfunction in vitro.

### 3.2. The Effect of IL-17A on BRB Integrity In Vivo

**Figure 2:**
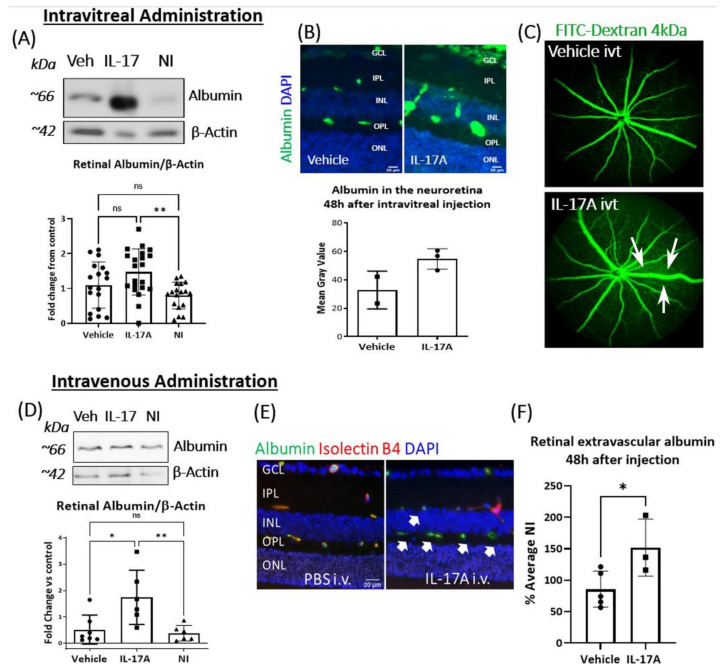
The effect of IL-17A in BRB integrity in vivo. Retinal albumin expression and albumin leakage were assessed 48 h after IL-17A intravitreal (itv, **A**–**C**) or intravenous (i.v., **D**–**F**) administration in *C57BL/6J* mice. (**A**) Western blot analysis of total albumin expression in the retina from vehicle (Veh, PBS) or IL-17A ivt, and control mice (no injection). Corresponding densitometry of albumin normalized to β-Actin. *n* ≥ 16 retinas from *n* ≥ 8 mice per group. (**B**) Albumin immunostaining (green) in retinal sections from IL-17A or PBS ivt mice. Scale bar = 25 µm. Graph showing mean gray value of albumin in the neuroretina (*n* ≥ 2 animals per group). (**C**) Representative images showing FITC-Dextran leakage from retinal vessels 48 h after IL-17A or PBS control ivt. White arrows: FITC-Dextran leakage, *n* ≥ 4 eyes per group. (**D**) Western blot analysis of total albumin expression in the retina from vehicle (Veh, PBS) or IL-17A i.v. and control mice (no injection); *n* ≥ 6 retinas from *n* ≥ 6 animals per group. (**E**) Representative images of extravascular albumin (green) outside Isolectin B4^+^ blood vessels (red) in the retina from PBS or IL-17A i.v. injected mice. White arrows: albumin leakage. (**F**) Quantification of extravascular albumin in the retina from vehicle or IL-17A i.v. injected mice. Blue—DAPI; *n* ≥ 3 eyes per group. Scale bar = 20 µm. Mean ± SD; ** *p* < 0.001, * *p* < 0.05 with One-Way ANOVA followed by Dunnett’s multiple comparisons in A and D and unpaired *t*-test in (**F**).

Wild-type *C57BL/6J* mice received IL-17A (ivt, 20 ng/µL/eye) or (i.v., 20 ng/g), and BRB function was examined 48 h later. Western blot showed significantly higher levels of albumin in the retinae from IL-17A ivt treated mice compared to mice that received no injection (NI) (Figure 2A). Retinal section immunostaining also showed higher levels of albumin expression in IL-17A ivt mice (Figure 2B). Micron IV examination revealed FITC-Dextran (4 kDa) extravasation in mice received IL-17A ivt injection compared to control PBS ivt mice (arrows, Figure 2C).

### 3.3. The Effect of IL-17A on pJAK1 Expression In Vitro and In Vivo

**Figure 3:**
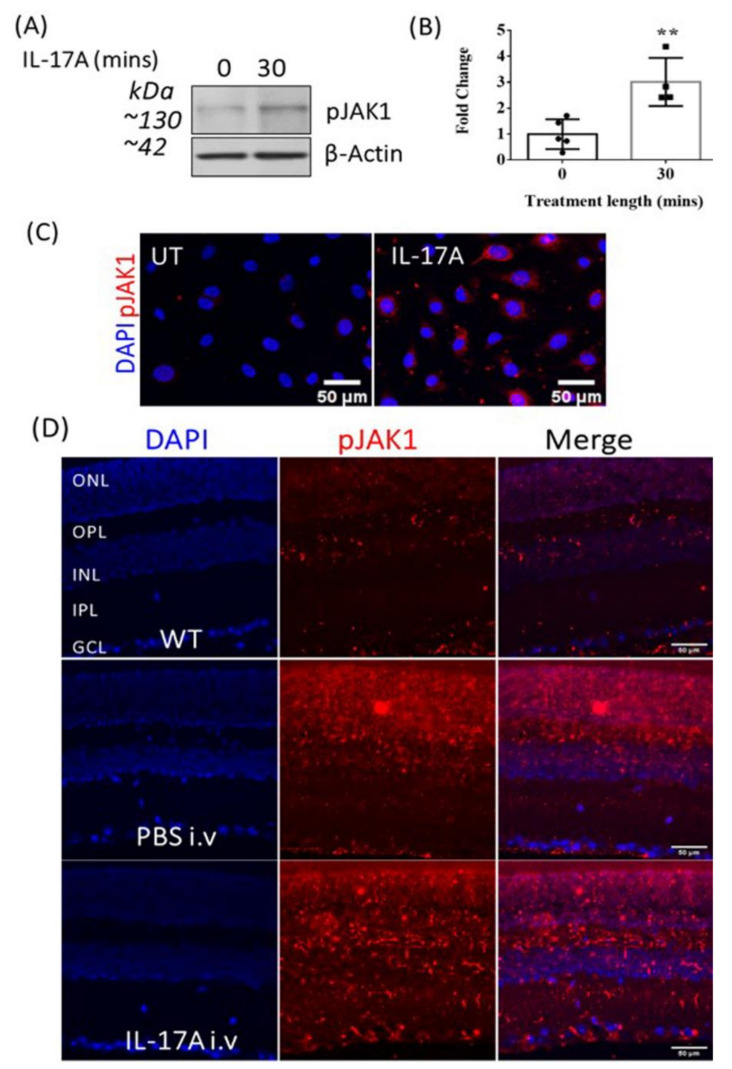
The effect of IL-17A on pJAK1 expression in bEnd.3 cells and the WT mouse retina. JAK1 phosphorylation was examined in bEnd.3 cells by Western blot and immunostaining, and in mouse retinal sections 48 h after IL-17A i.v. administration. (**A**) Representative Western blot and corresponding densitometry of pJAK1 in control (0 min) and IL-17A-treated bEnd3 cells. (**B**) Quantification of pJAK1 expression detected by Western blotting. Mean ± SD; ** *p* ≤ 0.01, unpaired *t*-test, *n* ≥ 4. (**C**) Representative images of pJAK1 immunocytochemistry in bEnd.3 cells from *n* ≥ 3 independent experiments. (**D**) Immunostaining of pJAK1 expression (red) in the murine retina 48 h after IL-17A or PBS i.v. injection compared to control non-injected wild-type (WT) mice, *n* ≥ 4.

**Figure A2:**
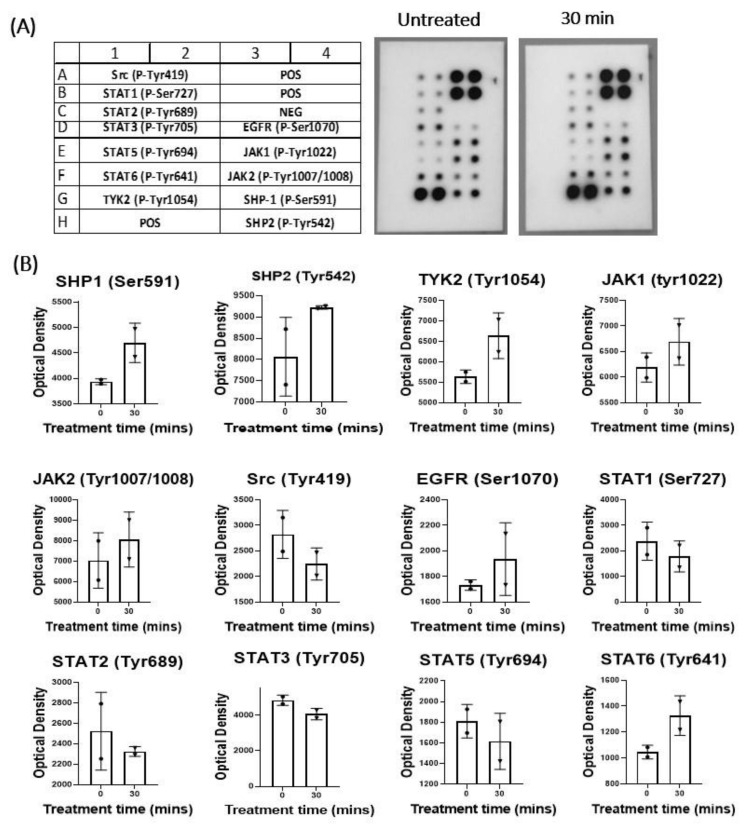
Array of JAK/STAT phosphorylation in ARPE19 cells in response to IL-17A treatment. Human ARPE-19 cells were treated with 50 ng/mL IL-17A for 30 min. Protein extracted from *n* = 4 independent experiments were pooled for array analyses, with one membrane per time point. (**A**) Array blot images from control and IL-17A treated group and blot identities were shown in the table. (**B**) Graphs showing the expression levels of the phosphorylated form of JAK/STAT family members.

**Figure A3:**
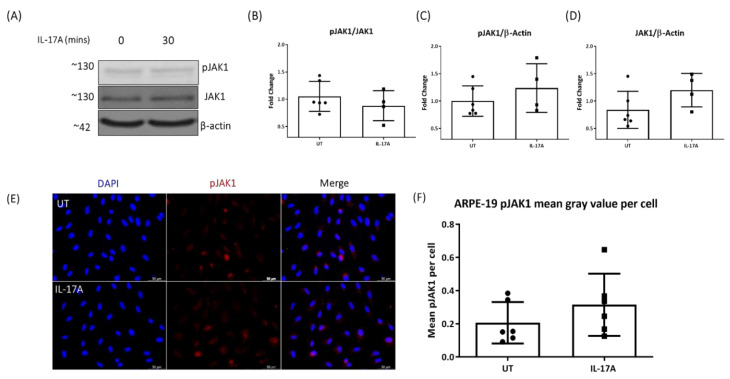
IL-17A does not significantly alter pJAK1 expression in ARPE-19 cells. Western blot (**A**) and corresponding (**B**–**D**) densitometry analysis of pJAK1, JAK1 and β-actin. (**E**) Representative immunostaining for pJAK1 (red) in control (UT) and IL-17A-treated ARPE19 cells, and (**F**) corresponding quantification. UT: untreated control.

**Figure A4:**
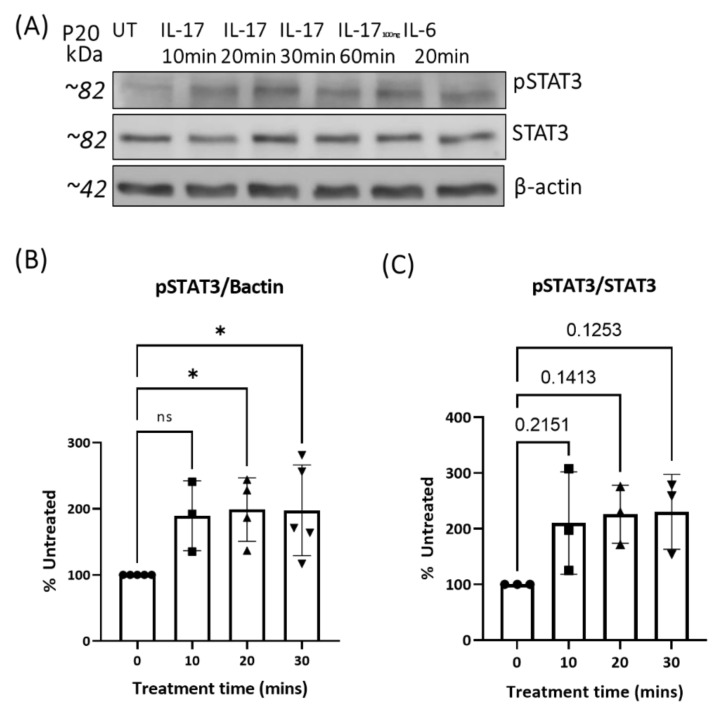
pSTAT3 expression in ARPE-19 cells after IL-17A treatment. (**A**) Representative Western blot images of *n* = 4 independent experiments. Densitometry of (**B**) pSTAT3/β-Actin and (**C**) pSTAT3/STAT3. Mean ± SD; *n* = 4; * *p* < 0.05 by One-Way ANOVA with Dunnett’s post hoc tests, ns: no statistical significance.

**Figure A5:**
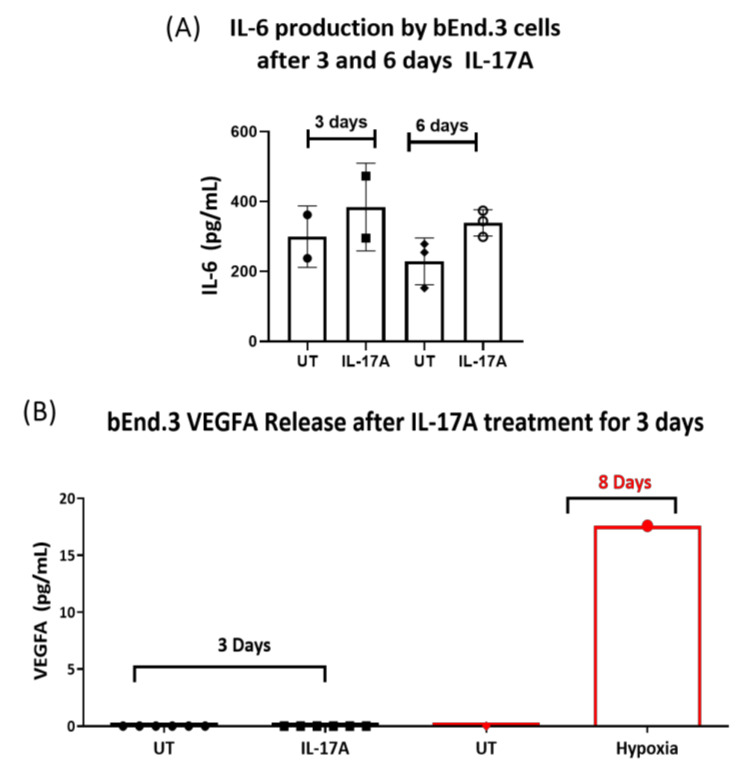
IL-17A may mediate release of IL-6, but not VEGFA, from bEnd.3 cells. Supernatants from control of IL-17A treated bEnd.3 cells were collected at relevant time points. The concentrations of IL-6 and VEGF were measured by ELISA. (**A**) The levels of IL-6 in bEnd.3 cell supernatant after 3 and 6 days of treatment with IL-17A. Mean ± SD, *n* = 2. (**B**) The levels of VEGFA in the supernatants from bEnd.3 cells after 3 days IL-17A treatment (black dots). Supernatants from bEnd.3 cells exposed to hypoxia (1% oxygen) for 8 days (red dots) were used as a positive control. Each point is representative of one technical replicate. Mean ± SD. UT, untreated; IL-17A, treated with 100 ng/mL of IL-17A.

To understand the signaling pathways involved in IL-17A-induced BRB damage, we investigated the JAK/STAT pathway, as it is a master regulator of cytokine receptor signaling. As the commercially available JAK/STAT array kit was only for human tissues, we conducted a human JAK/STAT array assay in IL-17A-treated ARPE19 cells. The results show that 30 min treatment with IL-17A increased the expression of SHP1, pJAK1, pTYK2 and pSTAT6 in ARPE19 cells (Appendix A Figure A2). Further Western blot and immunocytochemistry showed that IL-17A treatment significantly upregulated pJAK1 expression in bEnd.3 cells (Figure 3A–C) but not in ARPE19 cells (Appendix A Figure A3). However, pSTAT3 expression was increased in IL-17A-treated ARPE19 cells (Appendix A Figure A4). As IL-6 and VEGF can also active the JAK/STAT pathway, we measured their concentrations in the supernatants from control and IL-17A-treated bEnd.3 cells. We found that the 3–6 days of IL-17A treatment appeared to upregulate IL-6, but not VEGFA production (Appendix A Figure A5).

Injection (i.v.) of IL-17A upregulated pJAK1 expression in mouse retina, particularly the inner layers (ganglion cell layer and inner plexiform layer, Figure 3D). The location of pJAK1 positivity in the inner retina and the fact that in vitro treatment of ARPE19 cells (oBRB) with IL-17A did not seem to increase pJAK1 expression (Figure A3) led us to focus our study on the role of JAK1 in IL-17A-mediated iBRB damage, rather than oBRB damage.

### 3.4. JAK1 Inhibitor Tofacitinib Citrate Ameliorates IL-17A-Mediated Leakage in an In Vitro Model of iBRB

**Figure 4:**
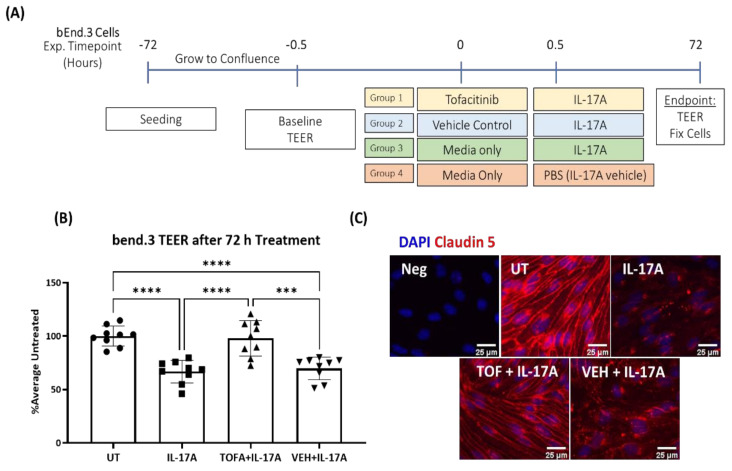
The effect of Tofacitinib Citrate on IL-17A-induced barrier dysfunction and claudin-5 junction dysmorphia in bEnd.3 cells. The bEnd.3 cells were pretreated with Tofacitinib Citrate (4.955 µM) for 30 min, followed by IL-17A (100 ng/mL) incubation for 30 min. TEER and claudin-5 expression in bEnd.3 cells was examined 72 h later. (**A**) Scheme of experimental design. (**B**) TEER in bEnd.3 cells from different groups. UT, untreated; Mean ± SD; *** *p* < 0.001, **** *p* ≤ 0.0001 by One-Way ANOVA with Tukey’s multiple comparisons test. (**C**) Immunostaining for claudin-5 (red), DAPI (blue). Scale bar = 25 µm.

**Figure A6:**
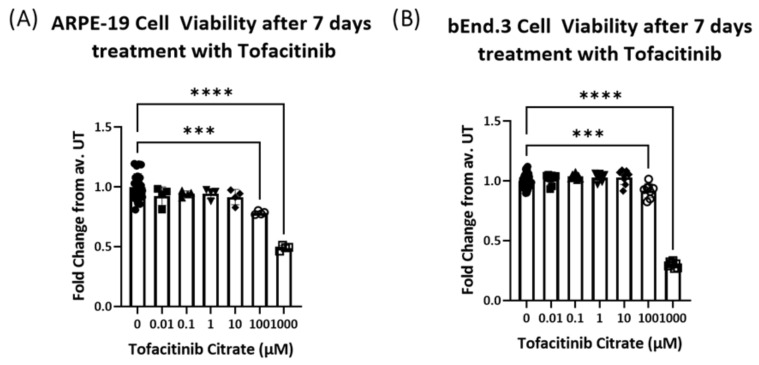
The effect of Tofacitinib Citrate on the viability of ARPE19 and bEnd.3 cells. The ARPE19 or bEnd.3 cells were treated with different concentrations of Tofacitinib for 7 days. Cell viability was evaluated by using the Alamar blue assay. (**A**) Viability fold change in ARPE-19 cells. (**B**) Viability fold change in bEnd.3 cells. *** *p* ≤ 0.0005, **** *p* ≤ 0.0001. One-Way ANOVA and Dunnett’s multiple comparisons test.

To further explore the role of JAK1 activation in IL-17A-mediated iBRB dysfunction, we tested the effect of a JAK1/3 inhibitor Tofacitinib Citrate in the in vitro model of iBRB, i.e., bEnd.3 cultured in transwell membrane (Figure 4A). Tofacitinib Citrate did not affect bEnd.3 cell viability at or below 4.955 µM (Appendix A Figure A6). Pretreatment of iBRB with Tofacitinib (4.955 µM) for 30 min significantly prevented IL-17A-mediated TEER reduction (Figure 4B) and preserved claudin-5 expression (Figure 4C).

### 3.5. The Effect of Tofacitinib Citrate in IL-17A-Mediated Retinal pJAK1 Expression

**Figure 5:**
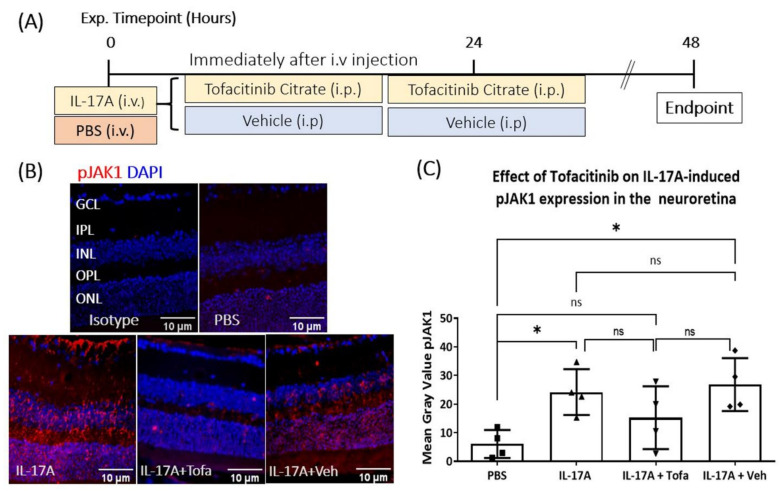
The effect of Tofacitinib Citrate on IL-17A-induced retinal pJAK1 expression. *C57BL/6J* mice received IL-17A or vehicle (PBS) i.v. injection were treated with or without Tofacitinib Citrate twice. Retinae were collected 48 h later and processed for immunostaining of pJAK1. (**A**) Schematic of experimental design. (**B**) Representative images of pJAK1 expression in retinas of mice that received PBS, IL-17A, IL-17A + Tofacitinib Citrate or IL-17A + vehicle control; *n* ≥ 5 animals per group. (**C**) Quantification of pJAK1 integrated density. Two or three images from the central retina were quantified per animal. Values were normalized to PBS control on the same slide. Mean ± SD. ROUT outliers test 2% was used to remove outliers. Cleaned data were compared by using One-Way ANOVA with Tukey’s multiple comparisons test, * *p* < 0.05; ns: no statistical significance.

Having shown that i.v. injection of IL-17A upregulated pJAK1 expression in mouse retina (Figure 3A), we moved to investigate if the upregulation of pJAK1 could be reduced by JAK1/3 inhibitor Tofacitinib Citrate. IL-17A- or vehicle-injected mice were administered with Tofacitinib Citrate (15 mg/kg) once a day, for two days, and retinal pJAK1 expression was evaluated 48 h later (Figure 5A). The results confirmed the upregulation of pJAK1 by IL-17A (Figure 5B,C), Similar levels of pJAK1 expression were present in IL-17A and IL-17A+vehicle groups. Tofacitinib Citrate treatment slightly reduced IL-17A-mediated retinal pJAK1 expression; however, the reduction did not reach statistical significance (Figure 5C), which may be related to the large variation and insufficient sample size.

### 3.6. The Effect of Tofacitinib Citrate on IL-17A-Mediated BRB Leakage In Vivo

**Figure 6:**
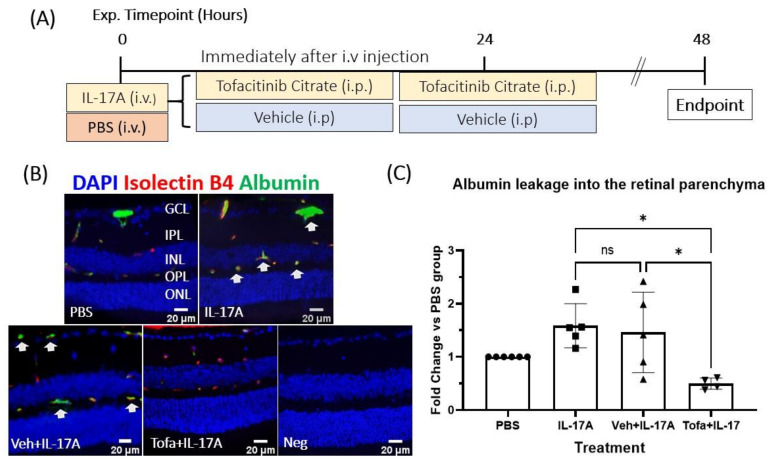
Tofacitinib Citrate ameliorates IL-17A-induced leakage in wild-type mice. C57BL/6J mice were treated with or without Tofacitinib Citrate immediately after IL-17A or vehicle (PBS) intravenous (iv) injection and were treated with Tofacitinib/vehicle again 24 h later. Retinae were collected 48 h later and processed for immunostaining of albumin and isolectin B4. (**A**) Schematic of experimental design. (**B**) Representative images showing albumin (green) and isolectin B4 (red, marker of vascular endothelial cells). Albumin outside the isolectin B4^+^ area was considered indicative of vascular leakage. White arrows: albumin leakage. (**C**) Quantification of albumin extravasation. Two or three images from the central retina were quantified per animal. Values were normalized to PBS control on the same slide. Mean ± SD; *n* ≥ 5 animals per group; ROUT outliers test 2% was used to remove outliers. Cleaned data were compared by using One-Way ANOVA with Tukey’s multiple comparisons test, * *p* < 0.05; ns: no statistical significance.

To understand the functional role of JAK1 activation in IL-17A-mediated iBRB dysfunction, mice were treated with Tofacitinib immediately after IL-17A injection, and again 24 h later (Figure 6A). Retinal vascular leakage was evaluated 48 h later by immunostaining of albumin. Extravascular albumin was successfully reduced in Tofacitinib Citrate–treated animals compared to those treated with vehicle control and IL-17A, or IL-17A alone (Figure 6B,C), indicating that JAK1 is functionally involved in iBRB leakage in IL-17A-induced BRB dysfunction.

## 4. Discussion

In this study, we showed that IL-17A can induce BRB dysfunction both in vitro and in vivo. Mechanistically, IL-17A damages the structure of tight junctions in both endothelial and RPE cells. In endothelial cells, the barrier destructive effect of IL-17A was mediated by activating the JAK1 signaling pathway. We further found that the JAK1 antagonist Tofacitinib Citrate could effectively ameliorate IL-17A-mediated BRB leakage. Tofacitinib is an FDA-approved anti-inflammatory medication, used to treat autoimmune diseases such as rheumatoid arthritis [49] and ulcerative colitis [51,52,53]. Our results suggest that Tofacitinib or other JAK1 inhibitors may be re-purposed for the management of IL-17A mediated macular oedema.

IL-17A is produced predominantly by CD4^+^ Th17 cells in response to cytokine IL-23 [54,55,56] and plays an important role in various autoimmune diseases, including uveitis [43,46,57]. Some innate immune cells, such as neutrophils [58,59,60] and γδT cells [61,62], can also produce IL-17A. In addition to its role in activating various immune cells, IL-17A is also reported to be involved in barrier dysfunction, including the blood–brain barrier [63]. In the retina, IL-17A is known to be involved in the pathogenesis of autoimmune uveitis [41,42,43,64], AMD [40,46,47,48,65,66,67,68] and DR [35,36,37,38,69]. Higher levels of IL-17A have been detected in the vitreous fluid of DMO patients [44,69], PDR patients [44,70], in AMD lesions [47] and in the serum of uveitis patients [42]. We found that both intravitreal and intravenous administration of IL-17A-induced BRB leakage, suggesting that IL-17A may damage the BRB from both the luminal and abluminal sides. The abnormal serum levels of IL-17A and intraocular IL-17A may all contribute to vascular leakage and macular oedema in patients with DMO, PDR, AMD and other retinal vascular diseases.

This study provides novel evidence of IL-17A-mediated pJAK1 activation in iBRB cells (vascular endothelial cells) and the murine retina. The signal transduction of several cytokines, including IL-17A, IL-6 and VEGF, have been reported to converge in the JAK/STAT pathway [71,72]. We found that 3–6 days of treatment with IL-17A could increase IL-6 but not VEGF production in bEnd.3 cells, indicating that IL-17A can active the JAK/STAT pathway directly and indirectly under chronic disease conditions, such as in DR and uveoretinitis. Many reports have delineated the role of the JAK/STAT pathway in retinal diseases involving BRB dysfunction, namely in DR [73,74], AMD [75,76] and uveoretinitis [77,78]. This finding is in agreement with studies which showed that Tofacitinib Citrate is effective in refractory anterior and intermediate uveitis and scleritis [79,80] and with another study, which showed it is protective against cytokine-mediated barrier dysfunction in the gastrointestinal tract [81]. Therefore, targeting the JAK/STAT pathway is a strategy that is worthy of exploration for the management of retinal diseases involving BRB dysfunction.

The limitations of our study include the use of cell lines ARPE-19 as oBRB and bEnd.3 as iBRB models, and that these models do not take into account of the effect of other supporting cells in the neurovascular unit, such as pericytes and Muller cells. Previous studies have shown that IL-17A can activate pericytes in vitro [82] through IL-17RA/C and induce endothelial basement membrane remodeling [83]. Future studies using primary or iPS-derived RPE or retinal endothelial cells together with pericytes and/or Muller cells in 3D culture models will be helpful to better understand the mechanism of IL-17A-induced BRB damage.

## 5. Conclusions

Our results suggest that IL-17A can damage the BRB through activation of the JAK1 signaling pathway. Since JAK1 is also involved in the signal transduction of other barrier-damaging cytokines, such as IL-6 and VEGF, targeting this pathway may be a novel approach for the management of macular oedema, particularly those who are resistant to anti-VEGF therapy.

## Figures and Tables

**Table 1:** Antibodies used for Immunostaining and Western blotting.

Target	Company, Product Number	Dilution Used
ZO-1	Thermo Fisher, 61-7300	1:50 (IF)
Claudin-5	Thermo Fisher, 34-1600	1:50 (IF)
Phospho-JAK1 (Tyr1034, Tyr1035)	Thermo Fisher, PA5-104554	1:50 (IF, IHC-P)
Albumin	Bethyl, a90-134a	1:800 (IHC-p), 1:1000 (WB)
Biotinylated Isolectin B4	Vector Labs, VEC.B-1205	1:50 (IHC-P)
Alexa Fluor^®^ 594 AffiniPure Donkey Anti-Rabbit IgG (H+L)	Stratech, 711-585-152	1:300 (IF), 1:300 (IHC-p)
Donkey Anti-Rabbit 488	Thermo Fisher, 34-1600	1:50 (IF)
Streptavidin, Alexa Fluor™ 594 conjugate	Thermo Fisher, S11227	1:300 (IHC-p)
Rabbit Anti-Mouse IgG H&L (HRP)	Abcam, ab6728	1:5000 (WB)
Alexa Fluor^®^ 488 AffiniPure Donkey Anti-Goat IgG (H+L)	Stratech, 705-545-147	1:300 (IHC-p)

IF, immunofluorescence; IHC-p, immunohistochemistry-paraffin; WB, Western blot.

## Data Availability

The data presented in this study are all contained within the main body of this article.
